# Protein aggregation and membrane lipid modifications under lactic acid stress in wild type and *OPI1* deleted *Saccharomyces cerevisiae* strains

**DOI:** 10.1186/s12934-016-0438-2

**Published:** 2016-02-17

**Authors:** Nadia Maria Berterame, Danilo Porro, Diletta Ami, Paola Branduardi

**Affiliations:** Department of Biotechnology and Biosciences, University of Milano-Bicocca, Piazza della Scienza 2, Milan, 20126 Italy; SYSBIO – Centre of Systems Biology, Milano and Roma, Italy; Department of Physics, University of Milano-Bicocca, Piazza della Scienza 3, Milan, 20126 Italy; Consorzio Nazionale Interuniversitario per le Scienze fisiche della Materia (CNISM) UdR Milano-Bicocca, Milan, 20126 Italy

**Keywords:** *Saccharomyces cerevisiae*, Lactic acid, Cell membrane, FTIR, Protein aggregation, *OPI1*

## Abstract

**Background:**

Lactic acid is a versatile chemical platform with many different industrial applications. Yeasts have been demonstrated as attractive alternative to natural lactic acid producers since they can grow at low pH, allowing the direct purification of the product in the desired acidic form. However, when very high concentrations of organic acids are reached, the major limitation for a viable production is the toxic effect of the product. The accumulation in the cytosol of H^+^ and of the weak organic counter-anions triggers a cellular reprogramming. Here, the effects of lactic acid exposure on *Saccharomycescerevisiae* have been evaluated by Fourier transform infrared (FTIR) microspectroscopy. In addition to -omic techniques, describing these responses in terms of systems and networks, FTIR microspectroscopy allows a rapid acquisition of the cellular biochemical fingerprint, providing information on the major classes of macromolecules.

**Results:**

FTIR analyses on *Saccharomyces cerevisiae* cells under lactic acid stress at low pH revealed some still uncharacterized traits: (1) a direct correlation between lactic acid exposure and a rearrangement in lipid hydrocarbon tails, together with a decrease in the signals of phosphatidylcholine (PC), one of the main components of cell membrane; (2) a rearrangement in the cell wall carbohydrates, including glucans and mannans (3) a significant yet transient protein aggregation, possibly responsible for the observed transient decrease of the growth rate. When repeated on the isogenic strain deleted in *OPI1*, encoding for a transcriptional repressor of genes involved in PC biosynthesis, FTIR analysis revealed that not only the PC levels were affected but also the cell membrane/wall composition and the accumulation of protein aggregates, resulting in higher growth rate in the presence of the stressing agent.

**Conclusions:**

This work revealed novel effects evoked by lactic acid on cell membrane/wall composition and protein aggregation in *S. cerevisiae* cells. We consequently demonstrated that the targeted deletion of *OPI1* resulted in improved lactic acid tolerance. Considering that stress response involves many and different cellular networks and regulations, most of which are still not implemented in modelling, these findings constitute valuable issues for interpreting cellular rewiring and for tailoring ameliorated cell factories for lactic acid production.

**Electronic supplementary material:**

The online version of this article (doi:10.1186/s12934-016-0438-2) contains supplementary material, which is available to authorized users.

## Background

In the last decades, with the advent of recombinant DNA technologies, and more recently with the implementations of synthetic biology [1], the use of microorganisms for the production of fuels and chemicals is moving from possible to real [2]. Indeed, microorganisms offer a tremendous potential as cell factories, both for the production of natural as well as recombinant products, and among them yeasts combine the advantage of unicellular state with a eukaryotic organization [3].

Among the variety of products that can be obtained from microbial factories, lactic acid emerges as a versatile chemical platform with many different industrial applications [4–10]. Engineered yeasts can represent a valuable alternative to the natural producers, the lactic acid bacteria (LAB), since they can grow at low pH. In this condition, the organic acid is mainly in its undissociated form, readily usable for polymerization to poly-lactic acid (PLA), a biodegradable bioplastic that has already entered in the market [11]. Another main advantage related to the ability of yeasts to grow at relative low pH is that it is dramatically reduced the use of salt (CaCO_3_) and, therefore, the subsequent removal of by-products (CaSO_4_). In spite of the demonstrated capability of engineered *Saccharomyces cerevisiae* strains to produce lactic acid at high yield, production and productivity [12], the presence of considerable amount (up to 80 g/L) of product in the medium imposes a high degree of stress to the cells, very likely impairing their further potential. Indeed, since the lipophilic undissociated form of the acid in the medium mainly permeates the plasma membrane by simple diffusion, once in the near-neutral cytosol the chemical dissociation of the weak acid occurs, leading to the release of protons and of the respective counter-anions that accumulate within the cell. This process causes several cell alterations. Briefly, on the one hand the accumulation of H^+^ causes an intracellular acidification. This in turn triggers a number of alterations, such as the decrease of DNA and RNA synthesis rate, the inhibition of metabolic activities and, in extreme cases, the disruption of the proton gradient across the plasma membrane. On the other hand, the accumulation of weak acid counter-anions, according to their specific characteristics, may lead to an increase in turgor pressure, oxidative stress, protein aggregation, lipid peroxidation, inhibition of membrane trafficking, and perturbation of plasma and vacuolar membranes spatial organization, reviewed in [13]. In literature, the accumulation of lactate is described to have a pro-oxidant effect [14, 15], to cause a strong impact on iron metabolism [16], to promote vacuolar fragmentation and to impair intracellular amino-acid homeostasis [17]. These reports, together with others describing the effects of diverse organic acids, point out that the evoked responses are organic acid-dependent and involving the cell as a whole. Therefore, techniques that can depict and describe the cell as an entire system at the macromolecular level can be of help in our need of understanding how microbial factories react to productions and of designing how to possibly tailor them for improving performances.

Together with -omic techniques, other approaches can be used complementarily. In particular, Fourier transform infrared (FTIR) spectroscopy is a non-invasive technique that allows rapid acquisition of the biochemical fingerprint of the sample under investigation, giving information on the content and structure of the major biomolecules, including lipids, proteins, carbohydrates and nucleic acids [18–22]. Moreover, FTIR microspectroscopy, obtained by coupling an infrared microscope to the FTIR spectrometer, makes it possible to collect the IR spectrum from a selected sample area down to ~20 μm × 20 μm, requiring therefore a limited amount of sample. Here we describe the application of FTIR microspectroscopy to characterize *S. cerevisiae* intact cells challenged with lactic acid at low pH.

By applying this technique, a direct correlation between exposure to lactic acid and decrease of phosphatidylcholine (PC), one of the most abundant membrane phospholipids, has been observed. Together with that, we detected a significant protein aggregation, likely responsible for the observed decrease of the growth rate in the initial phase of growth.

Opi1 is a transcriptional repressor of genes involved in PC biosynthesis [23]. Hypothesizing a pivotal role of membrane rearrangement in triggering cellular response, we further investigated the effect of lactic acid in *OPI1* deleted cells, finding that indeed in these cells the growth delay is less pronounced.

From these results, modifications of membrane composition and protein aggregation emerge as novel responses evoked by lactic acid exposure, suggesting that novel targets involved in membrane anabolism and protein turnover can be considered both for interpreting cellular rewiring and for tailoring ameliorated lactic acid producing cell factories.

## Results

### Analysis of the FTIR absorption spectrum of *Saccharomyces cerevisiae* cells

**Fig. 1:**
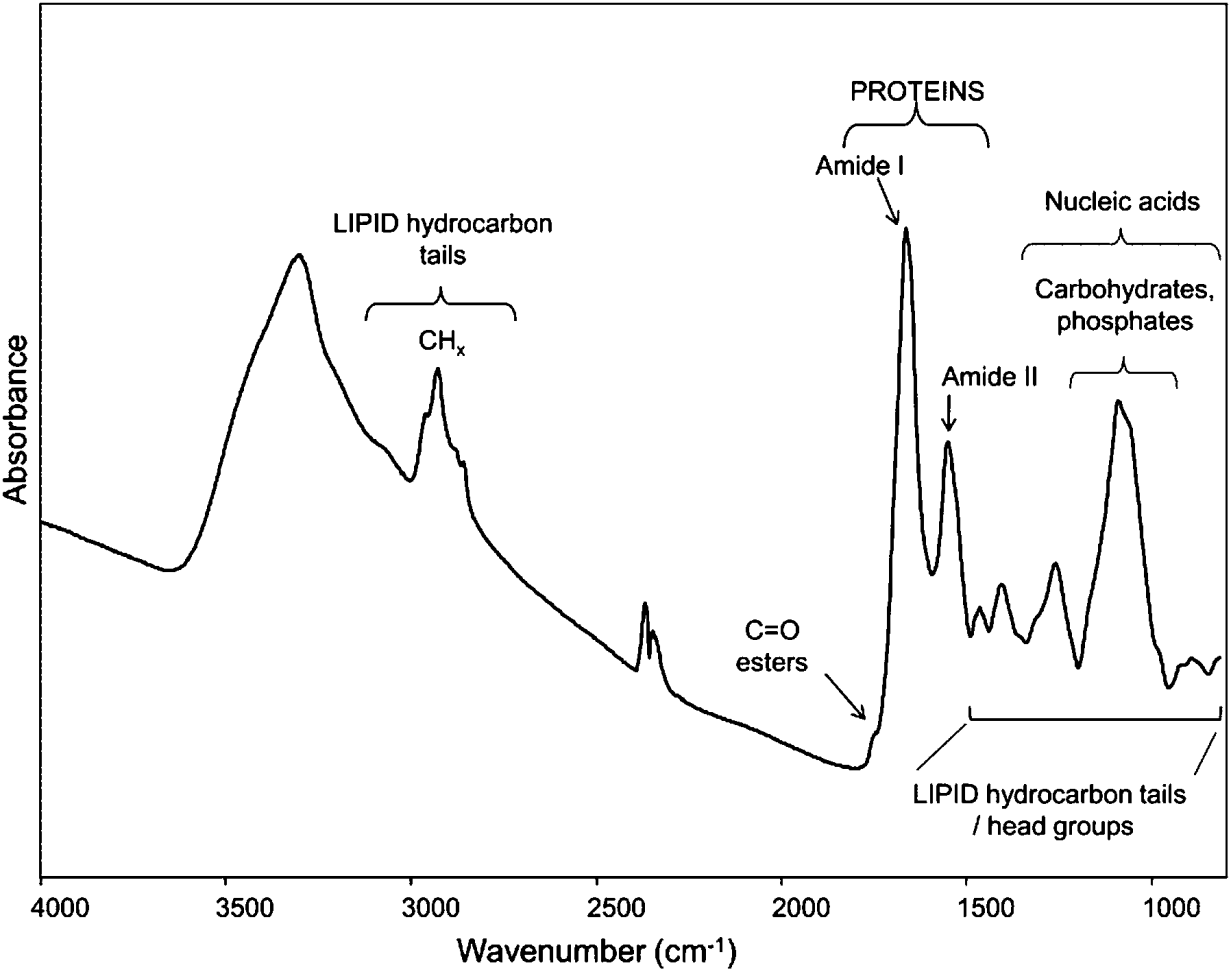
FTIR spectrum of *S. cerevisiae* intact cells. FTIR absorption spectrum of BY4741 strain cells, grown in shake flasks in minimal (YNB) medium with 2 % w/v glucose. FTIR analysis was performed at 18 h after the inoculation, corresponding to the exponential phase of growth. The assignment of selected bands to the main biomolecules is reported

We have chosen to describe *S. cerevisiae* cells growing in the presence or absence of inhibitory concentrations of lactic acid through their IR absorption spectrum. To exemplify a possible outcome of this analysis and the potential of the consequent observations, in Fig. 1 the measured IR absorption spectrum of *S. cerevisiae* intact cells, collected during the exponential phase of growth on minimal glucose medium (Additional file 1: Figure S1), is reported.

As illustrated, the spectrum is complex since it results from the absorption of the different biomolecules. In particular, the lipid hydrocarbon tails absorb between 3050 and 2800 cm^−1^ and between 1500 and 1350 cm^−1^, where also lipid head group absorption occurs, while around 1740 cm^−1^ the ester carbonyl IR response is observed [22, 24]. In addition, between 1700 and 1500 cm^−1^ the spectrum is dominated by the amide I and amide II bands, respectively due to the C=O stretching and the NH bending of the peptide bond. In particular, the amide I band gives information on the protein secondary structure and aggregation [19, 25–28]. Furthermore, the spectral range between 1250 and 900 cm^−1^ is dominated by the absorption of phosphate groups mainly from phospholipids and nucleic acids, as well as by the C–O absorption of carbohydrates [20–22].

To better evaluate possible spectral changes occurring under stressful conditions, often imposed by the fermentation processes, the second derivatives of the FTIR absorption spectra have been analysed, as they enable to resolve the overlapping components of the IR absorption bands [29]. Therefore, the results presented in the next sections will directly report the second derivatives spectra of *S. cerevisiae* cells grown in the different media and collected at different times after inoculation.

### FTIR microspectroscopy analysis of *Saccharomyces cerevisiae* BY4741 strain under lactic acid stress

**Fig. 2:**
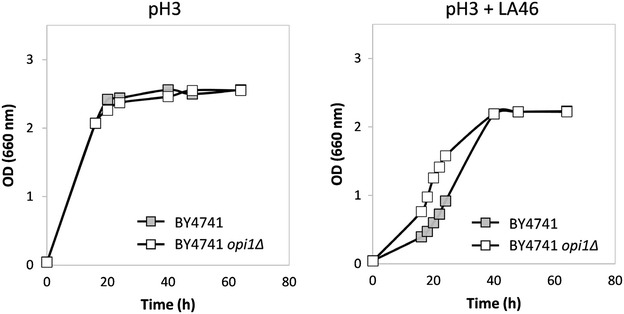
Growth of *S. cerevisiae* BY4741 and BY4741 *opi1Δ* strains in the absence and in the presence of lactic acid. Cells were grown in shake flasks in minimal (YNB) medium with 2 % w/v glucose without (*left panel*) or with (*right panel*) 46 g/L lactic acid at pH3. Growth was determined as OD at 660 nm. *Light grey full squares*: BY4741 strain. *Open squares*: BY4741 *opi1Δ* strain. The data reported here are representative of three independent experiments (variation < 3 %)

*S. cerevisiae* BY4741 cells were challenged with increasing concentrations of lactic acid, observing a gradual effect, from no perturbation of the kinetic of growth (data not shown) to detrimental effects, measured as a reduction in growth rate (see Fig. 2, closed symbols, minimal medium with 2 % w/v glucose in the absence -left- and in the presence -right- of 46 g/L lactic acid at pH 3). Independently from the media, cells reached the stationary phase of growth, but with a time delay and a reduced final biomass when treated with lactic acid. It is therefore relevant to analyse cellular response in this transition phase, especially in the view of a possible industrial process, where environmental fluctuations are inescapable, but undesirable if affecting microbial performances.

Samples collected at 18 and 40 h after inoculation, respectively corresponding to the exponential and the stationary phase of growth, were then analysed by FTIR microspectroscopy.

**Fig. 3:**
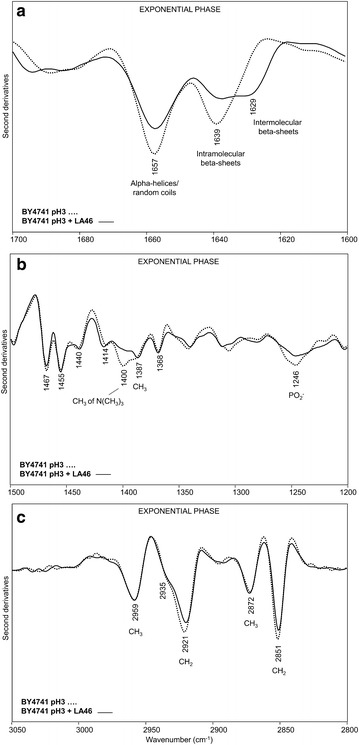
Second derivatives of the FTIR absorption spectra of *S. cerevisiae* BY4741 cells, in the absence and in the presence of lactic acid: exponential phase. Cells were grown in shake flasks in minimal (YNB) medium with 2 % w/v glucose in the absence and in the presence of 46 g/L of lactic acid (LA) at pH3. FTIR analysis was performed at 18 h after the inoculation, corresponding to the exponential phase of growth. **a** amide I band; **b** vibrational modes mainly due to lipid CH_2_/CH_3_ and to phosphate groups, **c**: stretching modes from lipid hydrocarbon tails. In **a**, **b** second derivative spectra have been normalized to the tyrosine band at ~1516 cm^−1^, while in **c** spectra have been normalized at the CH_3_ band at ~2959 cm^−1^

In Fig. 3 we reported the second derivative spectra of BY4741 *S. cerevisiae* cells grown for 18 h in the absence (pH3) and in the presence of 46 g/L of lactic acid at pH3 (pH3 + LA46), in the amide I band between 1700 and 1600 cm^−1^ (a), in the spectral ranges between 1500 and 1200 cm^−1^ (b) and between 3050 and 2800 cm^−1^ (c).

In the absence of the stressing agent the second derivative spectrum is characterized by a band at ~1657 cm^−1^, mainly due to alpha-helix and random-coil structures of the whole cell proteins, and by a band at ~1639 cm^−1^, assigned to intramolecular native beta-sheets [19, 26], (Fig. 3a). In the presence of lactic acid, an intensity reduction of the alpha helix/random coil and native beta-sheet components was observed, accompanied by the appearance of a new band at ~1629 cm^−1^, due to intermolecular beta-sheets, typical of protein aggregates [25, 27, 28, 30–32]. Interestingly, we found that the change in the intensity of the band assigned to protein aggregates is lactic acid dose-dependent (see Additional file 2: Figure S2a).

The spectral range between 1500 and 1200 cm^−1^ (Fig. 3b) is dominated by vibrational modes due to lipid hydrocarbon tails and head groups [22, 24]. In particular, the second derivative spectrum of cells grown in the absence of lactic acid is characterized by a number of well resolved bands mainly due to the CH_2_ and CH_3_ deformation modes: the ~1467 cm^−1^ band is due to the overlapping absorption of CH_2_ and CH_3_ [22, 24, 33], while the ~1455, 1440 and 1368 cm^−1^ bands are due to CH_3_ [22, 24], and the ~1414 cm^−1^ absorption to CH_2_ [34]. In addition, the component at ~1400 cm^−1^ is mainly assigned to the CH_3_ bending vibration of the N(CH_3_)_3_ head group of phosphatidylcholine (PC) and the absorption at ~1387 cm^−1^ can be assigned to the CH_3_ deformation mainly arising from ergosterol [22, 35, 36]. Finally, the component at ~1246 cm^−1^ is also observed, due to the PO_2_- stretching mode mainly from phospholipids and nucleic acids [20, 22].

In this study, we focused our attention on the bands that were found to significantly change after exposure to the stressing agent. In particular, the 1400 cm^−1^ and the 1246 cm^−1^ absorptions decreased in intensity concurrently when cells are in the presence of 46 g/L of lactic acid, indicating an overall reduction of PC component. Moreover, the ergosterol band at ~1387 cm^−1^ was found to become more resolved. We should also note that the variation of the PC marker band (~1400 cm^−1^) resulted again to be lactic acid dose-dependent (Additional file 2: Figure S2b).

Furthermore, in the spectral range between 3050 and 2800 cm^−1^ (Fig. 3c) the spectrum of cells grown at pH3 is characterized by four well resolved and intense bands due to the CH_2_ (at ~2921 and 2851 cm^−1^) and CH_3_ (at ~2959 and 2872 cm^−1^) stretching vibrations of lipid hydrocarbon tails [22, 24]. A shoulder around 2935 cm^−1^ is also present, that can be mainly assigned to the CH_2_ stretching of ergosterol [35].

Interestingly, in pH3 + LA46 cell spectrum, the CH_2_ stretching bands at ~2921 cm^−1^ and 2851 cm^−1^ were found to slightly decrease in intensity, likely suggesting a rearrangement of the hydrocarbon tails [37].

We investigated also the spectral range between 1200 and 900 cm^−1^ (see Additional file 3: Figure S3), dominated by the absorption of cell wall carbohydrates, including glucans and mannans [38]. As illustrated in Additional file 3: Figure S3a, compared to unchallenged cells, cells treated with lactic acid displayed a slight reduction in the intensity of the β1 → 3 glucan and mannan spectral components, accompanied by a weak but significant reduction of the low intensity band due to β1 → 6 glucans.

Overall, these results depict a change in the biochemical fingerprint of yeast cells exponentially growing in medium added with lactic acid. In particular, PC is not only one of the most abundant membrane phospholipids but it is also responsible for membrane fluidity [39, 40]. The decreasing in PC observed during the response to lactic acid exposure might therefore be a strategy adopted by the cells to make the membrane more compact and, consequently, less permeable to the lactic acid influx. As a consequence, this might also reflect in a general rearrangement of the transport rates. Moreover, if the plasma membrane is considered not only as a barrier between the extracellular and the intracellular environments, but also as a stress sensor [41], changes in its composition might additionally trigger a variety of intracellular events intended to rewire or adapt the cells to the different environment. As we will discuss in the next paragraph, the growth delay observed when cells are exposed to the stressing agent might be therefore related to the observed protein aggregation.

**Fig. 4:**
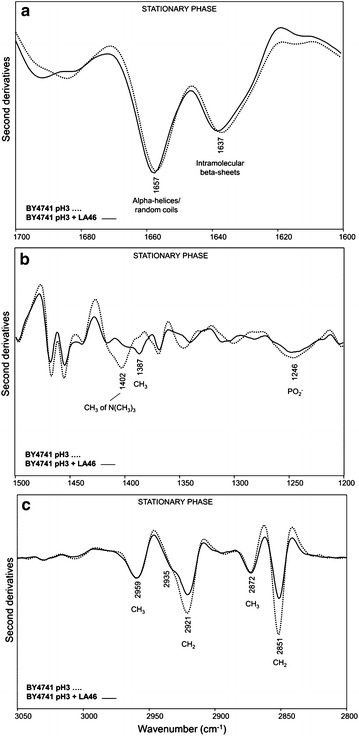
Second derivatives of the FTIR absorption spectra of *S. cerevisiae* BY4741 cells, in the absence and in the presence of lactic acid: stationary phase. Cells were grown in shake flasks in minimal (YNB) medium with 2 % w/v glucose in the absence and in the presence of 46 g/L of lactic acid (LA) at pH3. FTIR analysis was performed at 40 h after the inoculation, corresponding to the stationary phase of growth. a: amide I band; b: vibrational modes mainly due to lipid CH_2_/CH_3_ and to phosphate groups, **c**: stretching modes from lipid hydrocarbon tails. In **a** and **b** second derivative spectra have been normalized to the tyrosine band at ~1516 cm^−1^, while in **c** spectra have been normalized at the CH_3_ band at ~2959 cm^−1^

At 40 h after inoculation, corresponding to the stationary phase of growth, in the amide I band the spectral features of cells grown in the presence and in the absence of lactic acid resulted to be quite similar, with two main components at ~1657 cm^−1^ due to alpha helices and random coils, and at ~1637 cm^−1^ mainly due to intramolecular native beta-sheets (Fig. 4a). These results indicate that in this growth phase the lactic acid exposure does no more affect significantly the overall secondary structures of the whole cell proteins.

On the contrary, an important decrease of the PC marker band intensity at ~1402 cm^−1^ was still detected in pH3 + LA46 cells (Fig. 4b), accompanied by an increase of the ergosterol absorption at ~1387 cm^−1^ and a slight decrease of the PO_2−_ band at ~1246 cm^−1^. Furthermore, dramatic changes in the spectral features between 3050 and 2800 cm^−1^ were found. In particular, a significant intensity decrease of the CH_2_ bands at 2921 and 2851 cm^−1^, consistent with a reduction of the lipid hydrocarbon tail length, took place in cells exposed to lactic acid (Fig. 4c). Moreover, in agreement with the ergosterol absorption at ~1387 cm^−1^, the shoulder around 2935 cm^−1^ became more evident compared to pH3 cells. The analysis of the cell wall carbohydrate absorption between 1200 and 900 cm^−1^ (see Additional file 3: Figure S3b) highlighted firstly a higher level of β1 → 6 glucans in unchallenged cells at the stationary phase of growth, compared to the exponential. In addition, at 40 h after inoculation, in lactic acid treated cells we observed a reduction in intensity of the spectral components mainly due to glucans. These spectral changes, which suggest again a rearrangement of the cell wall properties, were found to be more pronounced in the stationary phase compared to the exponential (Additional file 3: Figure S3a).

### Effects of *OPI1* deletion on lactic acid tolerance and on macromolecular fingerprint

As described above, in the yeast strain under investigation a correlation between lactic acid exposure and a decrease in PC levels exists. Opi1p is a transcription factor that acts as a repressor of the genes involved in the synthesis of PC [23]. Consequently, we have envisaged *OPI1* as a useful target for further supporting this indication and, in particular, the effects of its overexpression and deletion were analysed under lactic acid stress. Since the *OPI1* gene overexpression caused severe growth deficiencies both in the absence and in the presence of lactic acid (data not shown), we focused our attention on its deletion. Figure 2 (open symbols) shows the growth curves obtained for the *OPI1*-lacking in the absence and in the presence of lactic acid. No remarkable differences were observed between the control and the *OPI1* deleted strain during growth without lactic acid at low pH (left panel), while lactic acid exerted a clear negative effect. Notably, in the limiting condition (right panel) a marked difference between the two strains was observed: the BY4741 *opi1Δ* rescued growth earlier than the parental strain, showing a faster growth rate (0.11 vs. 0.06 h^−1^) despite the two strains reached a similar final biomass value.

**Fig. 5:**
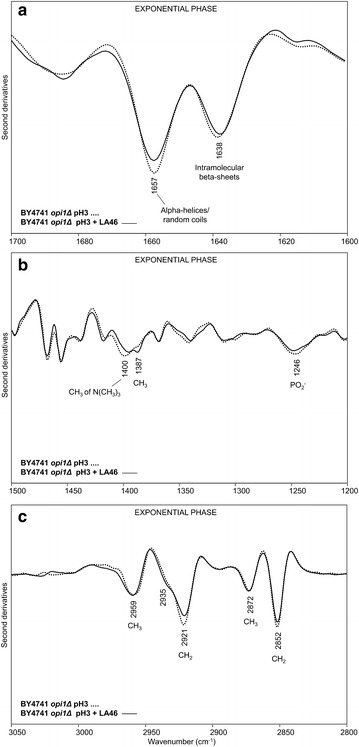
Second derivatives of the FTIR absorption spectra of *S. cerevisiae* BY4741 *opi1Δ* cells, in the absence and in the presence of lactic acid: exponential phase. Cells were grown in shake flasks in minimal (YNB) medium with 2 % w/v glucose in the absence and in the presence of 46 g/L of lactic acid (LA) at pH3. FTIR analysis was performed at 18 h after the inoculation, corresponding to the exponential phase of growth. **a** amide I band; **b** vibrational modes mainly due to lipid CH_2_/CH_3_ and to phosphate groups, **c**: stretching modes from lipid hydrocarbon tails. In **a** and **b** second derivative spectra have been normalized to the tyrosine band at ~1516 cm^−1^, while in **c** spectra have been normalized at the CH_3_ band at ~2959 cm^−1^

In Fig. 5, we reported the second derivative spectra of these cells collected in the exponential phase of growth (see also Additional file 4: Figure S4). In particular, in Fig. 5a the amide I band analysis indicates that—contrary to what observed for the parental strain (Fig. 3a)—the lactic acid exposure of the BY4741 *opi1Δ* cells did not dramatically affect the cell protein structures, just leading to a slight decrease in the intensity of the alpha-helix/random coil component at ~1657 cm^−1^, compared to unchallenged cells (Additional file 5: Figure S5a). Furthermore, a minor decrease of the PC marker band at ~1400 cm^−1^ and of the ~1246 cm^−1^ (PO_2−_) component occurred in pH3 + LA46 cells compared to pH3, accompanied by a slight increase of the ergosterol absorption at ~1387 cm^−1^ (Figs. 5b, 3b, Additional file 4: S4b and Additional file 5: S5b for comparison). In addition, a weak reduction in the intensity of the hydrocarbon tail CH_2_ absorption at ~2921 and ~2852 cm^−1^ (Fig. 5c) has been detected.

Moreover, for *opi1Δ* cells the spectral features mainly due to cell wall carbohydrates displayed in particular a slight decrease in the intensity of the β1 → 3 glucan bands upon LA treatment (see Additional file 3: Figure S3c). Indeed, the extent of these spectral variations was similar to that observed for the parental strain cells in the exponential phase (see Additional file 3: Figure S3a).

Overall, these results indicate that the *OPI1* deletion has a direct effect on the levels of PC, as expected, and this in turn avoids the formation of protein aggregates, as indicated by the absence of the aggregate marker band around 1629 cm^−1^ in the presence of lactic acid (Additional file 4: Figures S4a, Additional file 5: S5a, Additional file 6: S6a). This finally correlates with an increased tolerance to the stressing agent (Fig. 2).

**Fig. 6:**
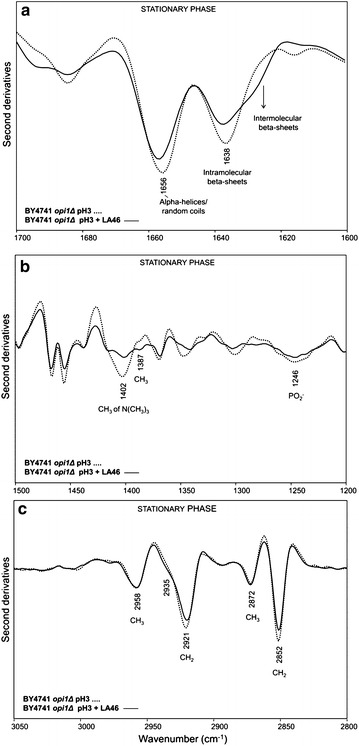
Second derivatives of the FTIR absorption spectra of *S. cerevisiae* BY4741 *opi1Δ* cells, in the absence and in the presence of lactic acid: stationary phase. Cells were grown in shake flasks in minimal (YNB) medium with 2 % w/v glucose in the absence and in the presence of 46 g/L of lactic acid (LA) at pH3. FTIR analysis was performed at 40 h after the inoculation, corresponding to the stationary phase of growth. **a** amide I band; **b** vibrational modes mainly due to lipid CH_2_/CH_3_ and to phosphate groups, **c** stretching modes from lipid hydrocarbon tails. In **a** and **b** second derivative spectra have been normalized to the tyrosine band at ~1516 cm^−1^, while in **c **spectra have been normalized at the CH_3_ band at ~2959 cm^−1^

Moreover, as reported in Fig. 6a, when BY4741 *opi1Δ* cells collected in stationary phase were examined, it appeared evident how the exposure to 46 g/L of lactic acid led to a decrease in intensity of both alpha-helix/random coil (~1656 cm^−1^) and intramolecular beta-sheet (~1638 cm^−1^) bands, accompanied by the appearance of a shoulder around 1629 cm^−1^, due to protein aggregates. Surprisingly, compared to pH3 cells, a significant decrease of the ~1402 cm^−1^ band was found, indicating a PC reduction in pH3 + LA46 cells (Fig. 6b). We should, however, note that the PC reduction in *opi1Δ* cells was slightly lower compared to that monitored for the lactic acid treated parental cells (see Fig. 4b, Additional file 6: S6b, Additional file 7: S7). In addition, in this phase of growth a weak intensity reduction of the CH_2_ bands between 3050 and 2800 cm^−1^ (Fig. 6c) was still observed for lactic acid treated *opi1Δ* cells.

Concerning the cell wall carbohydrate components (see Additional file 3: Figure S3d), upon LA treatment we observed spectral changes quite similar to those observed for the parental strain cells in the exponential phase (Additional file 3: Figure S3a). In addition, interestingly, the intensity of the β1 → 6 glucan band was again found to be higher in the unchallenged cells at the stationary phase, compared to the LA treated cells.

### Evaluation of unfolded protein response (UPR) under lactic acid exposure

As previously described, the growth advantage of BY4741 *opi1Δ* strain occurred during the exponential phase of growth (see Fig. 2). One of the main differences emerging from FTIR analysis is the phenomenon of protein aggregation, which in particular occurred at higher extent in the parental strain cells challenged with LA, compared to the *opi1Δ* strain (Figs. 3a, 5a, Additional file 5: S5a, Additional file 6: S6a) during this phase of growth.

Cells respond to the accumulation of unfolded proteins in the endoplasmic reticulum (ER) by the so-called unfolded protein response (UPR). UPR is triggered by the presence of protein aggregates, and involves a signal transduction cascade from the endoplasmic reticulum to the nucleus [42]. It acts at different levels, by promoting the transcription of genes encoding for chaperones localized in this cellular compartment, such as BiP (Hsp70) and PDI (Protein Disulfide Isomerase), by accelerating the rate of degradation of misfolded proteins with the action of ERAD (Endoplasmic Reticulum Associated protein Degradation) and by decreasing protein synthesis [43].

Because of the protein aggregation observed in exponentially growing cells under lactic acid stress, the UPR activation was evaluated for all the strains by monitoring *HAC1* mRNAs. Indeed, the transcription factor Hac1p is supposed to be the controller of the UPR in yeast. Cox and Walter [44] have identified two different forms of *HAC1* mRNAs: the full length (969 base pairs), which is present in cells whether or not the UPR is induced; the shorter one (generated by the splicing of 251 base pairs from the full length mRNA form) that appears only when the UPR is induced by Ire1p.

**Fig. 7:**
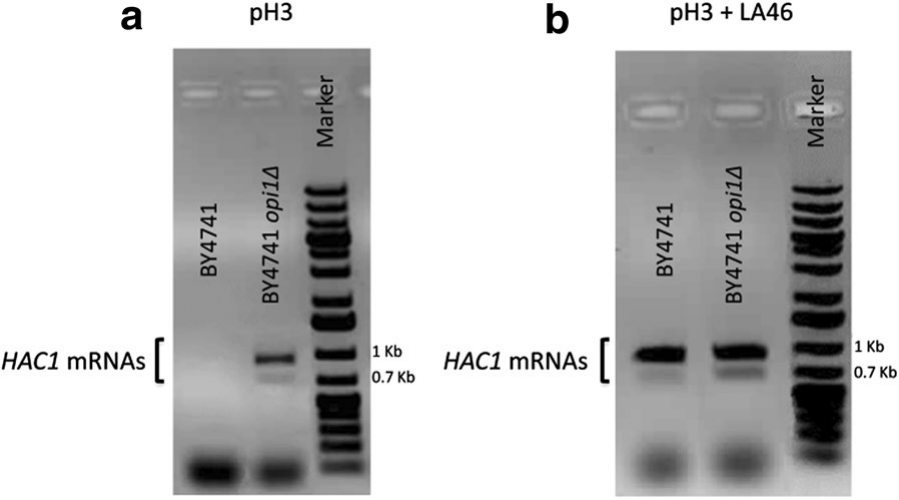
Evaluation of UPR in BY4741 and BY4741 *opi1Δ*. The *HAC1* mRNAs amplification was performed at 18 h after the inoculation, corresponding to the exponential phase of growth, in the BY4741 and BY4741 *opi1Δ* strains exposed (**b**) or not (**a**) to 46 g/L lactic acid at pH3

Samples of BY4741 and BY4741 *opi1Δ* cells grown as previously described were collected 18 h after inoculation, mRNAs were isolated and treated for RT-PCR experiment with the specific amplification of the *HAC1* cDNA (Fig. 7). In the presence of lactic acid (Fig. 7b), the full length and the spliced *HAC1* mRNA are evident, indicating that the UPR is active in both strains. In the control condition, at pH3 without lactic acid (Fig. 7a), the shorter mRNA form is present only in the BY4741 *opi1Δ* strain, suggesting that in this strain the UPR mechanism is active even without the presence of the stressing agent.

### Lactic acid and the triggering of lipid peroxidation

Lipid peroxidation is another of the reported effects of the weak organic counter-anions on *S. cerevisiae* cells [45], even if the triggering of this radical reaction was never reported for lactic acid exposure. Lipid peroxidation is a sudden molecular rearrangement that starts with the attack of a radical Reactive Oxygen Species (ROS) to a double bond of a polyunsaturated fatty acid, resulting in the formation of radical polyunsaturated fatty acids. These species, due to their high reactivity, can lead to the formation of several products including malondialdehyde (MDA), which can be, therefore, used as an index of lipid peroxidation level.

**Fig. 8:**
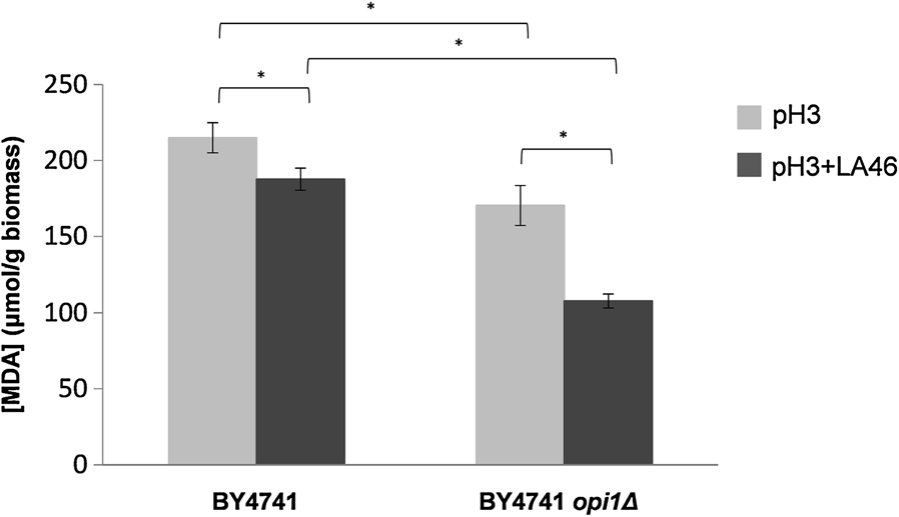
Evaluation of lipid peroxidation for cells stressed with lactic acid. Cells were grown in minimal medium until the exponential phase was reached and then they were treated or not with a pulse of lactic acid 46 g/L at pH 3. After 30 min the cells were collected and the levels of MDA were evaluated. *Dark grey bars* cells shocked with LA. *Light grey bars* control. *p ≤ 0.05 Student’s *t* test

Here we were interested to determine if lipid peroxidation can occur after a sudden exposure to lactic acid. For this experiment, BY4741 and BY4741 *opi1Δ* cells were grown in minimal medium until the exponential phase was reached and then they were treated with a pulse of lactic acid (46 g/L at pH 3), and without the stressing agent at pH3 as control. After 30 min, cells were collected and the levels of MDA were evaluated (see “Methods”), (Fig. 8).

Unexpectedly, the presence of lactic acid correlates with a statistically significant decrease in peroxidized lipid content, phenomenon particularly pronounced in the deleted strain (13 and 37 % decrease for the BY4741 and BY4741 *opi1Δ,* respectively). In particular, in both tested conditions (with or without lactic acid stress) the peroxidized lipid content was statistically significant lower in the BY4741 *opi1Δ* strain compared to the parental strain (21 and 43 % decrease, respectively at pH3 and at pH3 with LA 46 g/L).

## Discussion

This study has been focused on the evaluation of *S. cerevisiae* response to lactic acid stress. In particular, we characterized BY4741 and the derived *OPI1* deleted strains during the exponential and the stationary phase of growth, in the presence and in the absence of lactic acid at a concentration that is not limiting, but significantly affecting cell growth. From the experiments here reported some novel descriptions of rearrangements due to lactic acid exposure on *S. cerevisiae* cells have emerged, mainly related to the lipid and protein profiles.

In BY4741 parental strain cells, during the exponential phase of growth (T18), the presence of lactic acid caused a rearrangement of lipid hydrocarbon tails and decreased the level of PC (Fig. 3b, c). Since PC is one of the main components of cell membranes directly involved in fluidity [39, 40], its decrease might be a strategy adopted by the cell to modify membrane permeability, and thus to counteract the lactic acid influx into the cytosol. A concurrent reduction of lipid peroxidation has been also observed. It can be speculated that unsaturated membrane lipids decreased in favour of saturated ones. This hypothesis is supported by literature data regarding the exposure of the yeast *Zygosaccharomyces bailii* to acetic acid [46]. *Z. bailii*, well known but still not fully characterised for organic acid tolerance, resulted to be able to induce profound rearrangements in the plasma membrane after acetic acid treatment. In particular, a decrease of glycerophospholipids—specifically of PC—in favour of sphingolipids has been described, together with an increase in the degree of lipid saturation. These events can merge in reducing membrane fluidity, increasing the order of its structure and, hence, rendering it less permeable to acetic acid. In our FTIR experiments, no significant variation of the lipid acyl chain unsaturation degree was observed, possibly due to a poor amount of total unsaturation in the yeast lipid composition [47].

Since plasma membrane is not only a barrier between the extracellular and the intracellular environment, but also an important stress sensor, changes in its composition, such as the decrease of PC levels and the rearrangement of lipid hydrocarbon tails, might trigger multiple intracellular cascades [41]. We cannot exclude that also protein aggregation (Fig. 3a), a process potentially responsible for decreasing the growth rate of the BY4741 strain (Fig. 2), is connected to the same regulatory network. This hypothesis is supported by the fact that several proteins involved in the signalling cascades interact with the plasma membrane [48]. Furthermore, PC is also a source of lipid signalling molecules, playing an important role in signal transduction [49].

During the stationary phase of growth (T40), in the *S. cerevisiae* cells exposed to lactic acid the rearrangement of membrane lipids, likely started during the exponential phase of growth (Fig. 3b, c), was more pronounced (Fig. 4b, c), possibly leading to an even more stable structure of the membrane, necessary to counteract the effect of lactic acid. Moreover, also the increase of ergosterol, compared to the unchallenged cells, might have a contribution on membrane rigidity [50]. Interestingly, we also found that lactic acid exposure affected the composition of the cell wall carbohydrates; in particular, a reduction of glucans was observed (Additional file 3: Figure S3b). Since, as reported in literature [51], plasma membrane is also involved in glucan, mannan and chitin synthesis, changes in the cell wall composition might be a consequence of the cell membrane rearrangement. Contrariwise, protein structure was no more affected by the presence of lactic acid in the stationary phase of growth (Fig. 4a). Therefore, we can speculate that the protein aggregation had been counteracted by the UPR, activated during the exponential phase of growth.

In the second part of the study it was highlighted how the deletion of *OPI1* can have pleiotropic effects on lactic acid stress response. Indeed, its deletion affected not only the PC levels, the direct gene target, but also the degree of lipid peroxidation and the accumulation of protein aggregates. More in detail, during the exponential phase of growth, the changes related to membrane components observed under lactic acid exposure in the parental strain (Fig. 3b, c) were not so pronounced in the BY4741 *opi1Δ* strain (see Figs. 5b, c, Additional file 5: S5b, c). Moreover, not significant protein aggregation was observed, again suggesting a correlation between the two rearrangements (Fig. 5a). The evaluation of the UPR activation supports the hypothesis that the absence of protein aggregates in the mutant strain might be due to the fact that these cells are ready to deal with stress. Indeed, in the BY4741 *opi1Δ* strain, and differently from the parental strain, the UPR appeared to be active not only upon lactic acid exposure but also in the control condition (minimal medium at pH3). On the contrary, the accumulation of protein aggregates in the parental strain might be due to the fact that UPR has to be *de novo* activated.

We should add that we cannot exclude that the changes in the composition of cell wall carbohydrates and lipids, as well as in UPR, mainly observed in the parental strain exposed to lactic acid, are also due to a reduced growth rate. However, in the *opi1Δ* strain the presence of lactic acid does not determine profound physiological changes despite reducing its growth rate, suggesting therefore a complex but specific responsive network.

All this considered, we can conclude that the effect of *OPI1* deletion is possibly only indirectly influencing lactic acid tolerance, but it discloses still unravelled mechanisms and networks of cellular responses. We might further speculate that in BY4741 protein aggregation is a strategy for switching off the current cellular setting and promoting a more effective rewiring. In the described experiments, a stressful but not limiting condition for growth was applied: it has to be mentioned that when more stringent conditions are imposed or naturally occur, cellular rewiring is mainly intended to preserve some individuals, with the consequence of promoting the entrance in stationary phase of growth or even more in cryptobiosis. This has obviously to be carefully evaluated when a process of production is developed, as in the case of different sporulating bacteria [52].

## Conclusions

This study pointed out that the exposure to lactic acid in *S. cerevisiae* results in profound changes, never elucidated in literature, at the plasma membrane, in terms of its compositions and oxidative damage, as well as at the cell wall, and intracellularly, in terms of protein aggregation.

Moreover, it was highlighted how the deletion of *OPI1* affected not only the PC levels, direct gene target, but also the lipid peroxidation and the accumulation of protein aggregates; these changes might contribute to the increased robustness of the BY4741 *opi1Δ* strain in the presence of lactic acid.

Here we showed that despite the high number of studies on lactic acid production and cellular responses, connections and regulations among cellular mechanisms and structures are still far from being elucidated. To this aim, FTIR microspectroscopy is a powerful tool to unravel emerging properties of the cellular systems that, together with other studies focused on depicting the networks of stress responses, can be of help in tailoring optimized bioprocesses.

## Methods

### Yeast strains, media and cultivation

The BY4741 and the BY4741 *opi1Δ* strains were obtained from EUROSCARF.

Yeast cultivations were performed in synthetic minimal medium (0.67 % w/v YNB Biolife without amino acids) with 2 % w/v d-glucose as carbon source, supplemented with leucine, uracil, methionine and histidine to a final concentration of 50 mg/L. Lactic acid stress was imposed by adding the desired amount of L-lactic acid (Sigma-Aldrich) to the culture medium. The final media were prepared starting from two different stock solutions, one of 100 g/L lactic acid and one of synthetic minimal medium 2X, to obtain the desired lactic acid concentration and maintaining the same concentrations of nutrients. The pH of the lactic acid and the culture media were adjusted to three with pellets of KOH and HCl 1M, respectively. Cell growth was monitored by measuring the OD at 660 nm at regular time intervals and cells were inoculated at an initial OD of 0.05. All cultures were incubated in shake flasks at 30 °C and 160 rpm and the ratio of flask medium volume was 5/1. For the lipid peroxidation experiment, exponentially growing cells were collected and transferred in flasks containing lactic acid 46 g/L, adjusted to pH 3. The cells were incubated at 30 °C and 160 rpm for 30 min.

### FTIR microspectroscopy

Yeast cells from BY4741 and BY4741 *opi1ΔS. cerevisiae* strains at 18 and 40 h of growth were washed three times in distilled water to eliminate medium contamination. Approximately 3 μL of the cell suspensions were then deposited onto an IR transparent BaF_2_ support, and dried at room temperature for at least 30 min to eliminate the excess water.

FTIR absorption spectra were acquired in transmission mode, between 4000 and 700 cm^−1^, by means of a Varian 610-IR infrared microscope coupled to the Varian 670-IR FTIR spectrometer (both from Varian Australia Pty Ltd), equipped with a mercury cadmium telluride (MCT) nitrogen-cooled detector. The variable microscope aperture was adjusted to ~100 μm × 100 μm. Measurements were performed at 2 cm^−1^ spectral resolution; 25 kHz scan speed, triangular apodization, and by the accumulation of 512 scan co-additions.

Second-derivatives spectra were obtained following the Savitsky-Golay method (third-grade polynomial, 9 smoothing points), after a binomial 13 smoothing points of the measured spectra [29], using the GRAMS/32 software (Galactic Industries Corporation, USA).

To verify the reproducibility and reliability of the spectral results, more than three independent sample preparations were analysed and, for each preparation, at least ten spectra for sample were measured.

In the Figures, reported data are representative of the independent experiments performed.

### Evaluation of UPR

Total RNA was extracted from cells in exponential growth phase (T 18 h) by the AurumTM Total RNA Mini Kit (BIO-RAD), following manufacturer’s instruction, and it was reverse transcribed by iScript™ cDNA Synthesis Kit (BIO-RAD), following manufacturer’s instruction.

#### HAC1 mRNAs amplification

The *S. cerevisiaeHAC1* mRNAs sequences were amplified by PCR using as a template the cDNA. Phusion^R^ High-Fidelity DNA polymerase (NEB no. M0530) was used on a GeneAmp PCR System 9700 (PE Applied Biosystem, Inc.). Standard conditions used were: 0.5 µM primers, 1 U of Phusion and 1.5 μL cDNA. The program used for amplification of mRNAs was as follows: after 30 s at 98 °C, 25 cycles (each cycle consisting of 7 s at 98 °C, 20 s at 62.6 °C and 30 s at 72 °C) were carried out, followed by 7 min at 72 °C. Oligonucleotides pairs for *HAC1* were as follows: HAC1_fw (5′-ATGGAAATGACTGATTTTGAACTAACTAG-3′) and HAC1_rev (5′-TCATGAAGTGATGAAGAAATCATTCAATTC-3′).

### Evaluation of lipid peroxidation

An estimation of lipid peroxidation was based on the level of malondialdehyde formed after lactic acid pulse stress of *S. cerevisiae* parental and deleted strains, as described in [53]. Briefly, after treatment with or without lactic acid the cells were collected, resuspended in 100 mM Tris pH 7.8 and broken by glass beads. After centrifugation the supernatant was collected and 250 µL of the extract were mixed with 500 µL of the mix TBARS (15 % w/v trichloroacetic acid, 0.375 % w/v thiobarbituric acid, 0.25 N hydrochloric acid). The solution was heated for 1 h in a boiling water bath. The absorbance of the sample was determined at 535 nm against a blank that contained all the reagents except the extract. Results were expressed as micromoles of malondialdehyde per gram of wet weight biomass.

## Additional files

10.1186/s12934-016-0438-2 Growth of *S. cerevisiae* BY4741 strain in minimal medium. Cells were grown in shake flasks in minimal (YNB) medium with 2 % w/v glucose. Growth was determined as OD at 660 nm. The data reported here is representative of three independent experiments (variation < 3 %).
10.1186/s12934-016-0438-2 Second derivatives of FTIR absorption spectra of *S. cerevisiae* BY4741 strain in the absence and in the presence of lactic acid. Cells were grown in shake flasks in minimal (YNB) medium with 2 % w/v glucose in the absence and in the presence of different concentration of lactic acid: pH3, 40 g/L and 46 g/L lactic acid (LA) at pH3. FTIR analysis was performed at 18 h after the inoculation, corresponding the exponential phase of growth. **a**: amide I band; **b**: vibrational modes mainly due to lipid CH_2_/CH_3_ and to phosphate groups. Derivative spectra have been normalized to the tyrosine band at ~ 1516 cm^−1^.
10.1186/s12934-016-0438-2 Second derivatives of the FTIR absorption spectra of *S. cerevisiae* BY4741 and *opi1Δ* cells, in the absence and in the presence of lactic acid: cell wall carbohydrate absorption between 1200 - 900 cm^−1^. Cells were grown in shake flasks in minimal (YNB) medium with 2 % w/v glucose in the absence (**a, b**) and in the presence of 46 g/L of lactic acid (LA) (**c, d**) at pH3. FTIR analysis was performed at 18 h (**a, c**) and at 40 h (**b, d**) after the inoculation, corresponding to the exponential and stationary phase of growth, respectively. Second derivative spectra have been normalized to the tyrosine band at ~ 1516 cm^−1^, for comparison. The assignment of selected bands to the main carbohydrate components of the cell wall is reported.
10.1186/s12934-016-0438-2 Second derivatives of the FTIR absorption spectra of *S. cerevisiae* BY4741 and *opi1Δ* cells, in the absence of lactic acid: exponential phase. Cells were grown in shake flasks in minimal (YNB) medium with 2 % w/v glucose at pH3. FTIR analysis was performed at 18 h after the inoculation, corresponding to the exponential phase of growth. **a**: amide I band; **b:** vibrational modes mainly due to lipid CH_2_/CH_3_ and to phosphate groups, **c**: stretching modes from lipid hydrocarbon tails. In **a** and **b** second derivative spectra have been normalized to the tyrosine band at ~ 1516 cm^−1^, while in **c** spectra have been normalized at the CH_3_ band at ~ 2959 cm^−1^.
10.1186/s12934-016-0438-2 Second derivatives of the FTIR absorption spectra of *S. cerevisiae* BY4741 and *opi1Δ* cells, in the presence of lactic acid: exponential phase. Cells were grown in shake flasks in minimal (YNB) medium with 2 % w/v glucose in the presence of 46 g/L of lactic acid (LA) at pH3. FTIR analysis was performed at 18 h after the inoculation, corresponding to the exponential phase of growth. **a**: amide I band; **b:** vibrational modes mainly due to lipid CH_2_/CH_3_ and to phosphate groups, **c**: stretching modes from lipid hydrocarbon tails. In **a** and **b** second derivative spectra have been normalized to the tyrosine band at ~ 1516 cm^−1^, while in **c** spectra have been normalized at the CH_3_ band at ~ 2959 cm^−1^.
10.1186/s12934-016-0438-2 Intensity variation of the protein aggregate and phosphatidylcholine (PC) marker bands. **a)** Intensity ratio between the protein aggregate IR band at 1629 cm^−1^ and the tyrosine peak at 1516 cm^−1^ for the parental (P) and *opi1Δ* strains, in the absence and in the presence of 46 g/L lactic acid (LA) at pH3, in the exponential and stationary phases of growth. The reported data were normalized to the sample with the maximum intensity. The error bars represent the standard deviation of the IR spectra measured in three independent experiments. The intensity of the peaks has been taken from second derivative spectra. **b**) The same procedure has been followed for the intensity ratio of the 1400 cm^−1^ band, IR marker band of PC.
10.1186/s12934-016-0438-2 Second derivatives of FTIR absorption spectra of *S. cerevisiae* BY4741 parental and *opi1Δ* strains: stationary phase. Cells were grown in shake flasks in minimal (YNB) medium with 2 % w/v glucose in the absence and in the presence of 46 g/L lactic acid at pH3. FTIR analysis was performed at 40 h after the inoculation, corresponding to the stationary phase of growth. Derivative spectra have been normalized to the tyrosine band at ~ 1516 cm^−1^.

## Abbreviations

FTIR: Fourier transform infrared
PC: phosphatidylcholine
LAB: lactic acid bacteria
PLA: poly-lactic acid
UPR: unfolded protein response
ROS: reactive oxygen species
MDA: malondialdehyde
