# Supplementary figures and images for: Protein aggregation and membrane lipid modifications under lactic acid stress in wild type and OPI1 deleted Saccharomyces cerevisiae strains
Source: Microb Cell Fact. 2016 Feb 17;15:39. doi: 10.1186/s12934-016-0438-2 (PMC4756461; doi:10.1186/s12934-016-0438-2)

## Slide 1
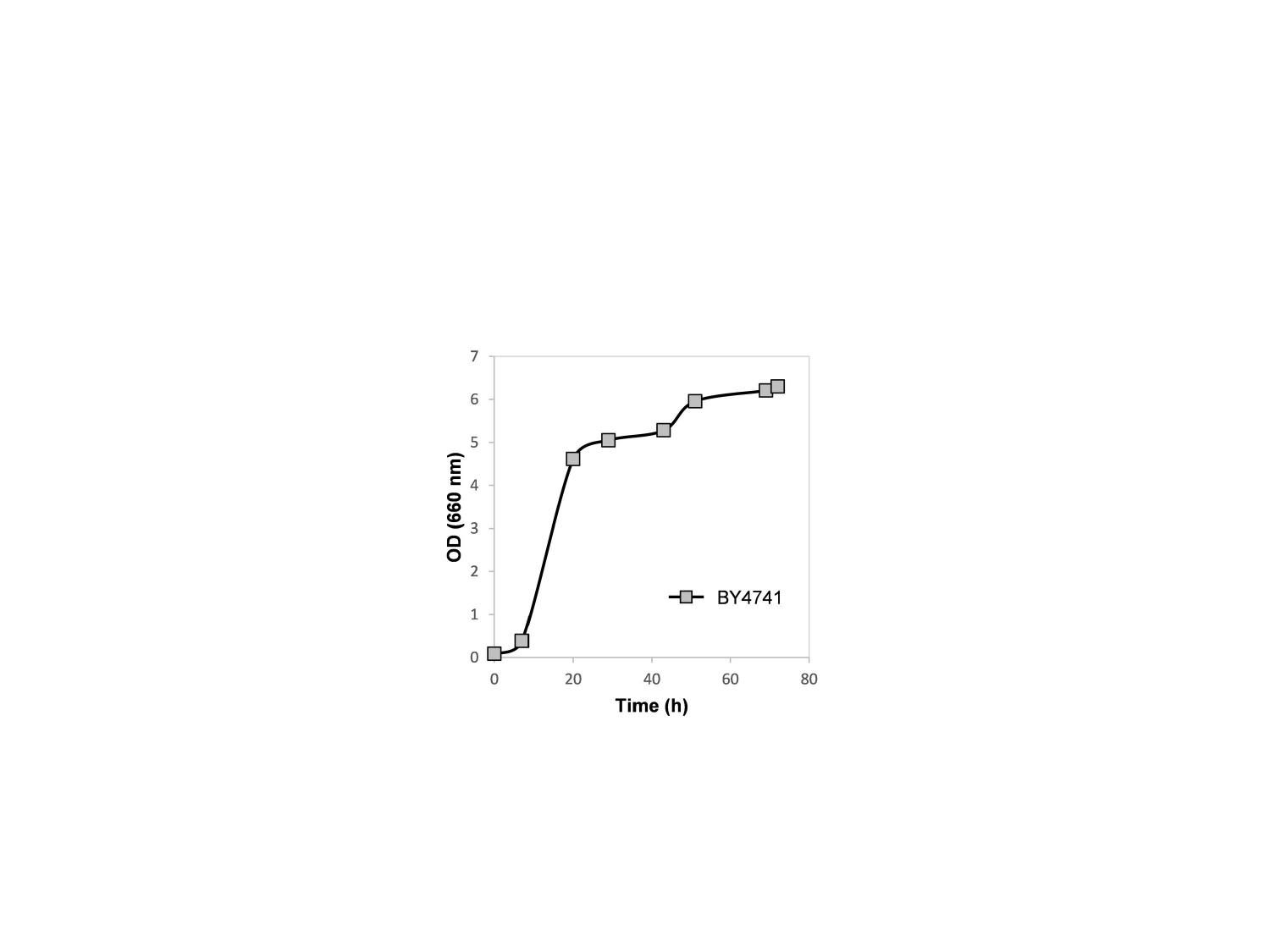

Supplement: Supplementary file 1 — 10.1186/s12934-016-0438-2 Growth of S. cerevisiae BY4741 strain in minimal medium. Cells were grown in shake flasks in minimal (YNB) medium with 2 % w/v glucose. Growth was determined as OD at 660 nm. The data reported here is representative of three independent experiments (variation < 3 %). [file 12934_2016_438_MOESM1_ESM.ppt]

## Slide 1
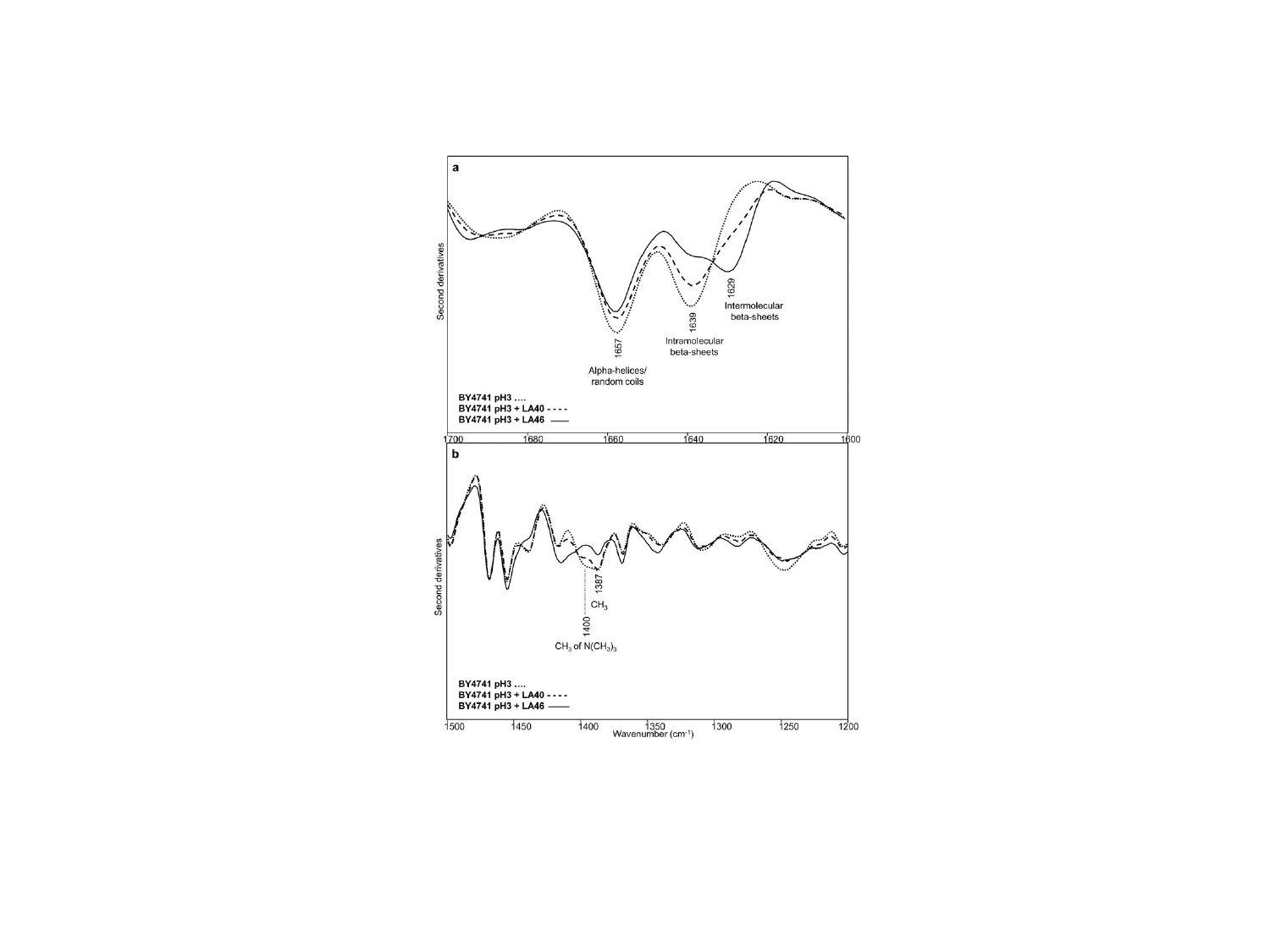

Supplement: Supplementary file 2 — 10.1186/s12934-016-0438-2 Second derivatives of FTIR absorption spectra of S. cerevisiae BY4741 strain in the absence and in the presence of lactic acid. Cells were grown in shake flasks in minimal (YNB) medium with 2 % w/v glucose in the absence and in the presence of different concentration of lactic acid: pH3, 40 g/L and 46 g/L lactic acid (LA) at pH3. FTIR analysis was performed at 18 h after the inoculation, corresponding the exponential phase of growth. a: amide I band; b: vibrational modes mainly due to lipid CH2/CH3 and to phosphate groups. Derivative spectra have been normalized to the tyrosine band at ~ 1516 cm−1. [file 12934_2016_438_MOESM2_ESM.ppt]

## Slide 1
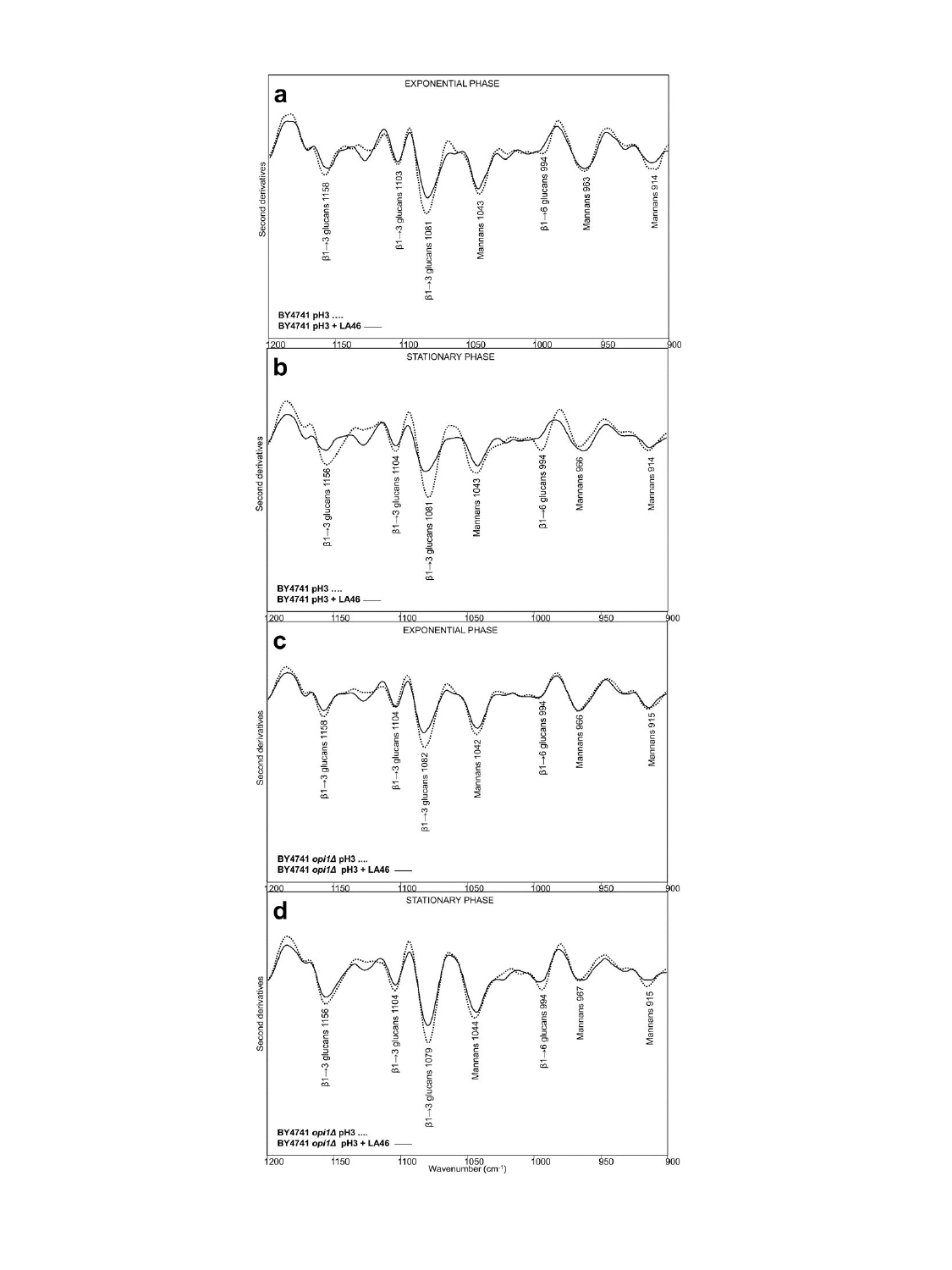

Supplement: Supplementary file 3 — 10.1186/s12934-016-0438-2 Second derivatives of the FTIR absorption spectra of S. cerevisiae BY4741 and opi1Δ cells, in the absence and in the presence of lactic acid: cell wall carbohydrate absorption between 1200 - 900 cm−1. Cells were grown in shake flasks in minimal (YNB) medium with 2 % w/v glucose in the absence (a, b) and in the presence of 46 g/L of lactic acid (LA) (c, d) at pH3. FTIR analysis was performed at 18 h (a, c) and at 40 h (b, d) after the inoculation, corresponding to the exponential and stationary phase of growth, respectively. Second derivative spectra have been normalized to the tyrosine band at ~ 1516 cm−1, for comparison. The assignment of selected bands to the main carbohydrate components of the cell wall is reported. [file 12934_2016_438_MOESM3_ESM.ppt]

## Slide 1
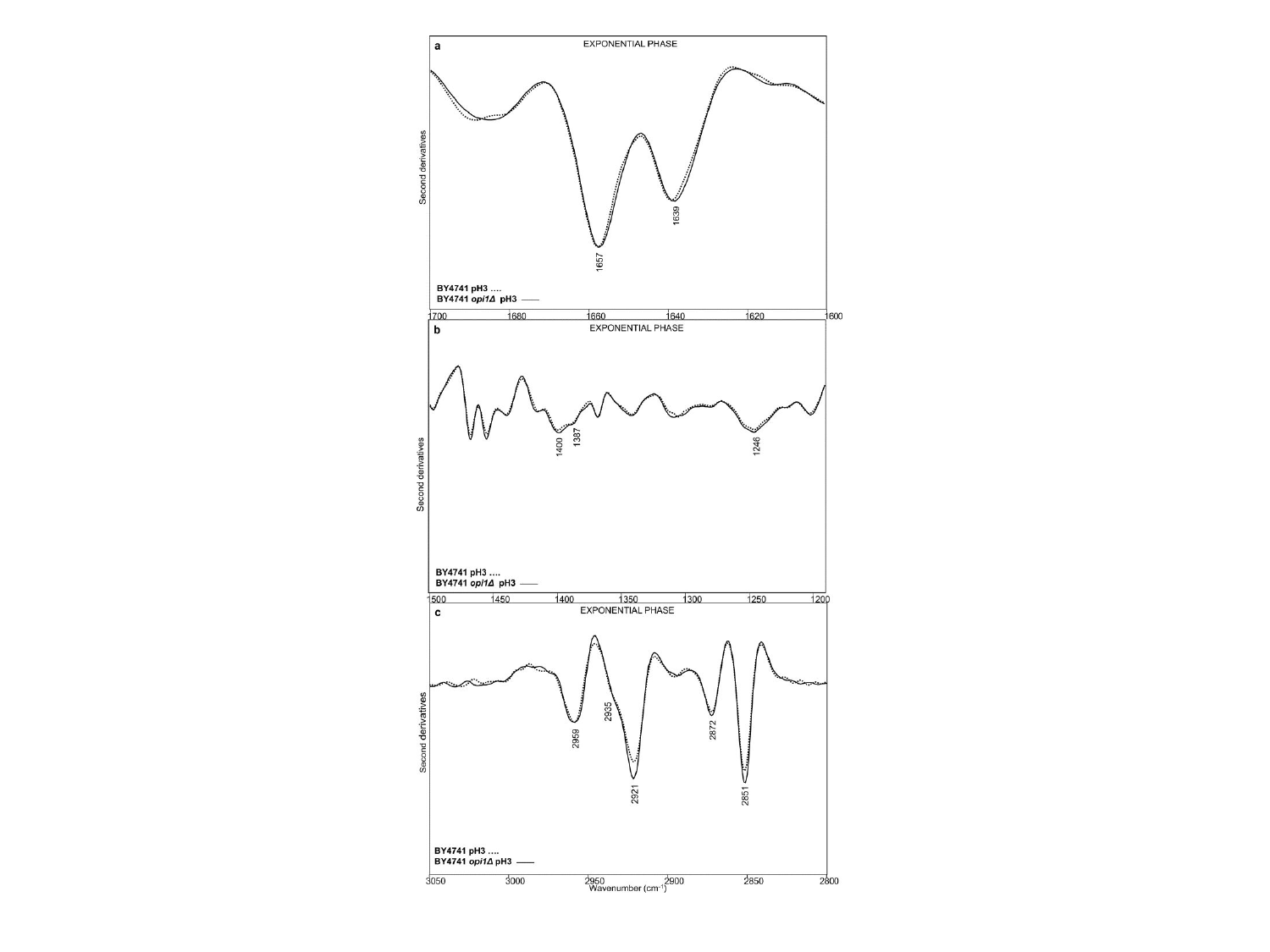

Supplement: Supplementary file 4 — 10.1186/s12934-016-0438-2 Second derivatives of the FTIR absorption spectra of S. cerevisiae BY4741 and opi1Δ cells, in the absence of lactic acid: exponential phase. Cells were grown in shake flasks in minimal (YNB) medium with 2 % w/v glucose at pH3. FTIR analysis was performed at 18 h after the inoculation, corresponding to the exponential phase of growth. a: amide I band; b: vibrational modes mainly due to lipid CH2/CH3 and to phosphate groups, c: stretching modes from lipid hydrocarbon tails. In a and b second derivative spectra have been normalized to the tyrosine band at ~ 1516 cm−1, while in c spectra have been normalized at the CH3 band at ~ 2959 cm−1. [file 12934_2016_438_MOESM4_ESM.ppt]

## Slide 1
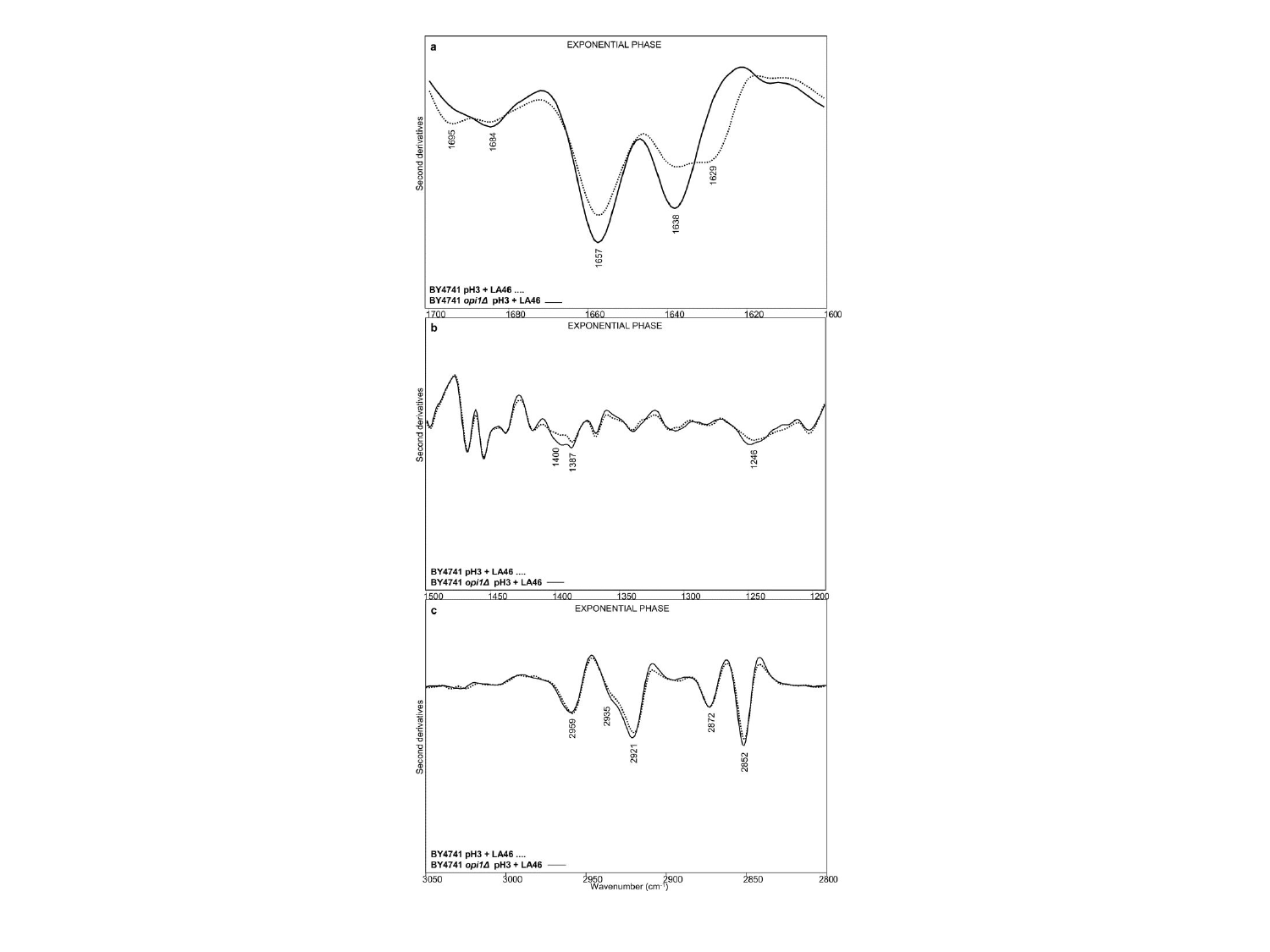

Supplement: Supplementary file 5 — 10.1186/s12934-016-0438-2 Second derivatives of the FTIR absorption spectra of S. cerevisiae BY4741 and opi1Δ cells, in the presence of lactic acid: exponential phase. Cells were grown in shake flasks in minimal (YNB) medium with 2 % w/v glucose in the presence of 46 g/L of lactic acid (LA) at pH3. FTIR analysis was performed at 18 h after the inoculation, corresponding to the exponential phase of growth. a: amide I band; b: vibrational modes mainly due to lipid CH2/CH3 and to phosphate groups, c: stretching modes from lipid hydrocarbon tails. In a and b second derivative spectra have been normalized to the tyrosine band at ~ 1516 cm−1, while in c spectra have been normalized at the CH3 band at ~ 2959 cm−1. [file 12934_2016_438_MOESM5_ESM.ppt]

## Slide 1
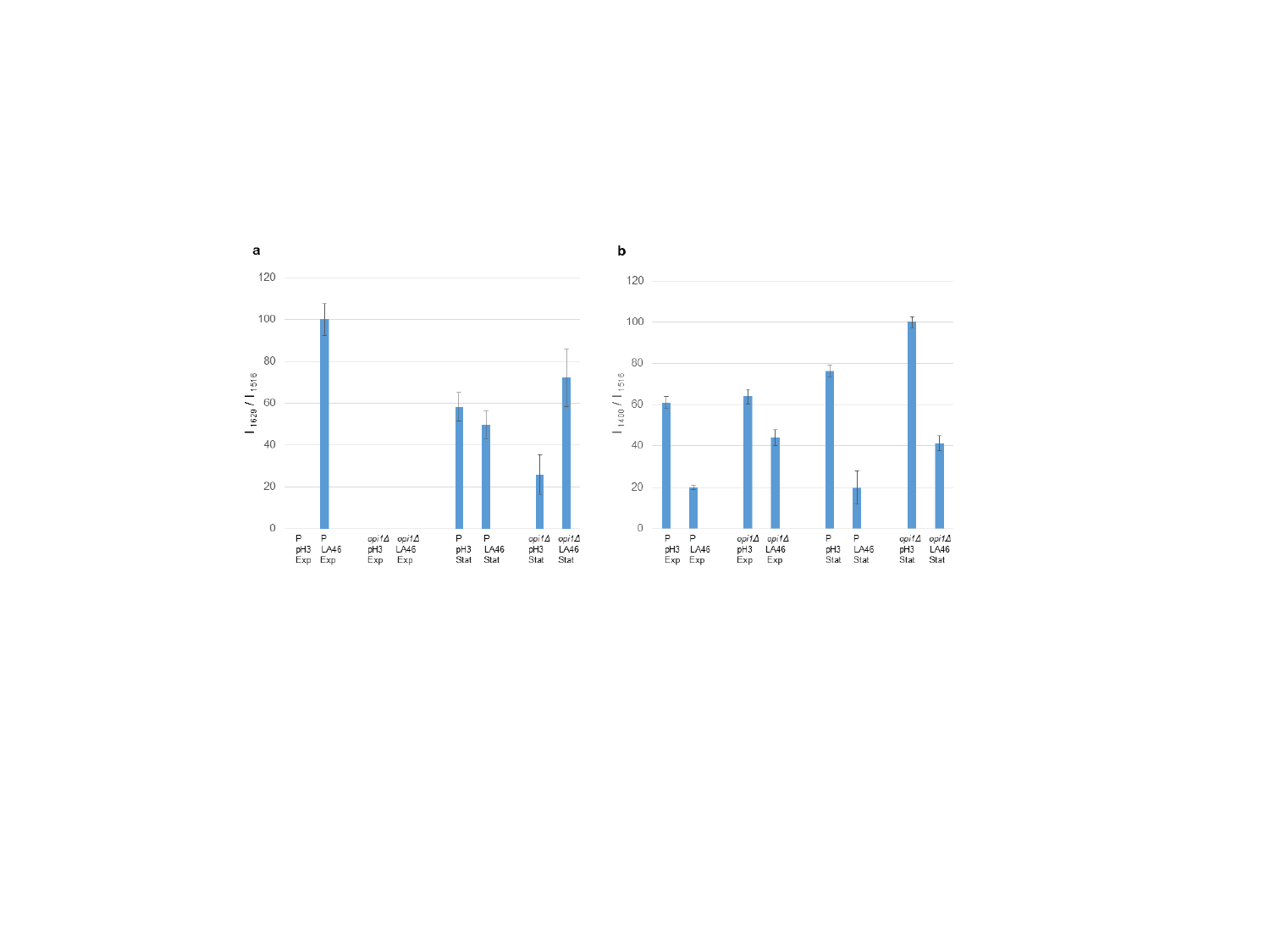

Supplement: Supplementary file 6 — 10.1186/s12934-016-0438-2 Intensity variation of the protein aggregate and phosphatidylcholine (PC) marker bands. a) Intensity ratio between the protein aggregate IR band at 1629 cm−1 and the tyrosine peak at 1516 cm−1 for the parental (P) and opi1Δ strains, in the absence and in the presence of 46 g/L lactic acid (LA) at pH3, in the exponential and stationary phases of growth. The reported data were normalized to the sample with the maximum intensity. The error bars represent the standard deviation of the IR spectra measured in three independent experiments. The intensity of the peaks has been taken from second derivative spectra. b) The same procedure has been followed for the intensity ratio of the 1400 cm−1 band, IR marker band of PC. [file 12934_2016_438_MOESM6_ESM.ppt]

## Slide 1
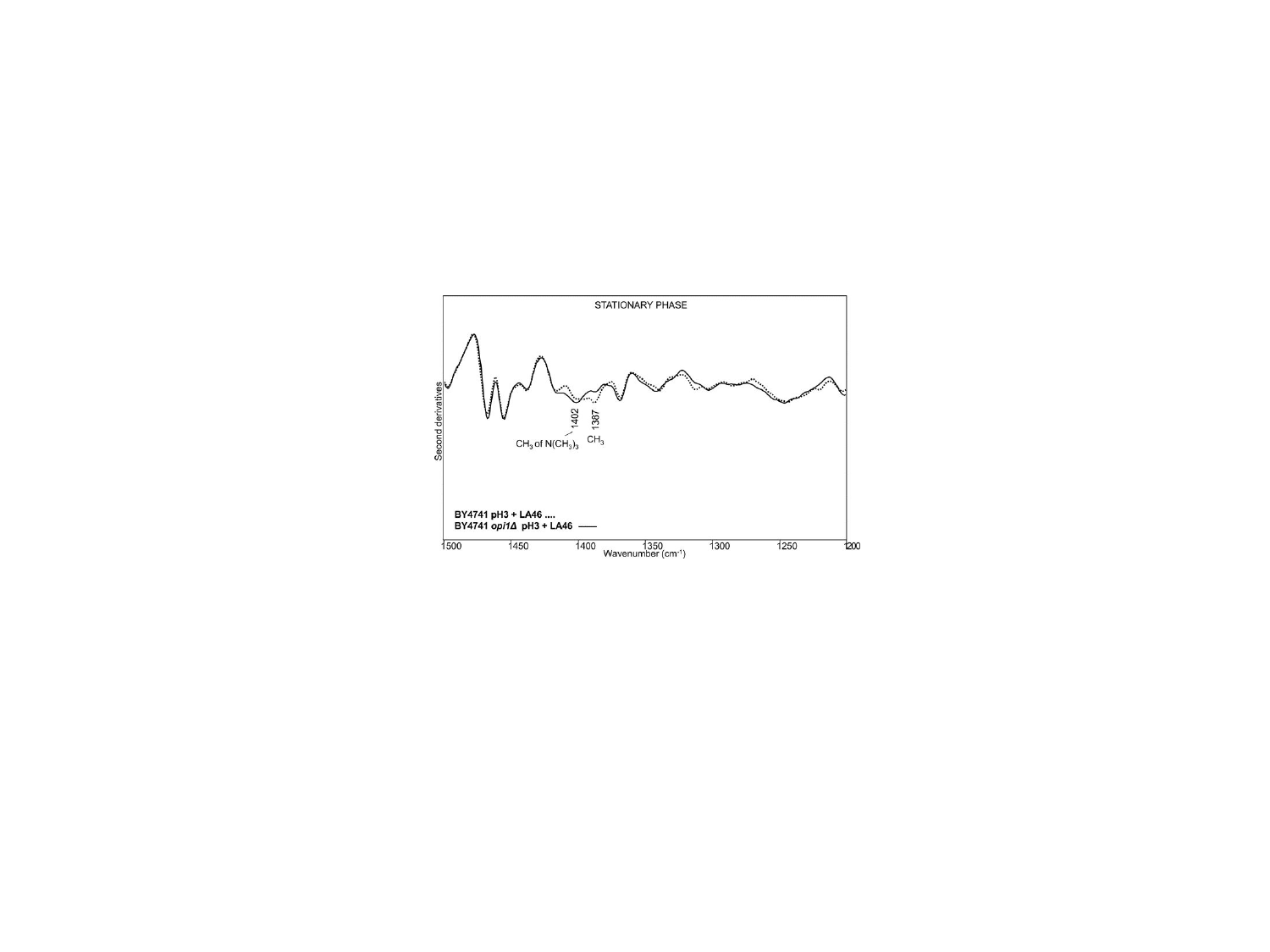

Supplement: Supplementary file 7 — 10.1186/s12934-016-0438-2 Second derivatives of FTIR absorption spectra of S. cerevisiae BY4741 parental and opi1Δ strains: stationary phase. Cells were grown in shake flasks in minimal (YNB) medium with 2 % w/v glucose in the absence and in the presence of 46 g/L lactic acid at pH3. FTIR analysis was performed at 40 h after the inoculation, corresponding to the stationary phase of growth. Derivative spectra have been normalized to the tyrosine band at ~ 1516 cm−1. [file 12934_2016_438_MOESM7_ESM.ppt]
